# A Cross-Site Intervention in Chinese Rural Migrants Enhances HIV/AIDS Knowledge, Attitude and Behavior

**DOI:** 10.3390/ijerph110404528

**Published:** 2014-04-23

**Authors:** Ning Li, Xiaomei Li, Xueliang Wang, Jin Shao, Juanhua Dou

**Affiliations:** 1 Department of Nursing, Health Science Center, Xi’an Jiaotong University, No.76, West Yanta Road, Xi’an 710061, Shaanxi, China; E-Mail: lining72@mail.xjtu.edu.cn; 2 Department of Public Health, College of Public Health, Health Science Center, Xi’an Jiaotong University, No.76, West Yanta Road, Xi’an 710061, Shaanxi, China; E-Mail: wangxl@mail.xjtu.edu.cn; 3 Department of Chinese Medicine, First Affiliated Hospital, Health Science Center, Xi’an Jiaotong University, No.277, West Yanta Road, Xi’an 710061, Shaanxi, China; E-Mail: szeraijin1228@126.com; 4 Xi’an Health School, Xi’an 710054, Shaanxi, China; E-Mail: doulady@126.com

**Keywords:** HIV/AIDS, cross-site joint prevention and control, rural migrants, China, knowledge attitude and behavior

## Abstract

*Background*: With the influx of rural migrants into urban areas, the spread of HIV has increased significantly in Shaanxi Province (China). Migrant workers are at high risk of HIV infection due to social conditions and hardships (isolation, separation, marginalization, barriers to services, *etc*.). *Objective*: We explored the efficacy of a HIV/AIDS prevention and control program for rural migrants in Shaanxi Province, administered at both rural and urban sites. *Methods*: Guidance concerning HIV/AIDS prevention was given to the experimental group (266 migrants) for 1 year by the center of disease control, community health agencies and family planning department. The intervention was conducted according to the HIV/AIDS Prevention Management Manual for Rural Migrants. A control group of migrants only received general population intervention. The impact of the intervention was evaluated by administering HIV/AIDS knowledge, attitudes and sexual behavior (KAB) questionnaires after 6 and 12 months. *Results*: In the experimental group; 6 months of intervention achieved improvements in HIV/AIDS related knowledge. After 12 months; HIV/AIDS-related knowledge reached near maximal scores. Attitude and most behaviors scores were significantly improved. Moreover; the experimental group showed significant differences in HIV-AIDS knowledge; attitude and most behavior compared with the control group. *Conclusions*: The systematic long-term cross-site HIV/AIDS prevention in both rural and urban areas is a highly effective method to improve HIV/AIDS KAB among rural migrants.

## 1. Introduction

The prevalence of the human immunodeficiency virus (HIV) infection in China is increasing each year, and the number of infected individuals reached 370,393 by the end of October 2010 [1]. Sexual intercourse is the main channel of HIV transmission [1]. Indeed, in China, 63.9% of HIV patients were infected through sexual intercourse, including 46.5% through sexual intercourse, and 17.4% through homosexual intercourse [2]. In China, about 80% of HIV-infected patients are from rural areas [3]. In Shaanxi, an economically important province in Western China, the rate of HIV infection has been rapidly rising. In 2010 alone, 462 new cases were identified, bringing the total prevalence in the province to 2,131 cases. Among these, 80% are between 20 and 49 years old, and most of them are from the low social classes, *i.e.*, farmers, migrant workers and unemployed citizens [4].

With the massive influx of rural population seeking work and business into urban areas, the numbers of rural migrants in large cities have rapidly increased. In 2010, the total number of migrants in China reached 221 million, of which about 72% were rural migrants. It is estimated that more than 3–5 million people will migrate from the countryside to cities and towns in the next 30 years in China [5]. The most recent statistics report that 1,851,017 people migrated to Shaanxi Province in 2009 [6]. These rural migrants have specific demographic and socioeconomic characteristics, such as lower educational levels, and residing a long distance from their hometown. In addition, these migrants suffer from a number of social conditions and hardships (isolation, separation from family, marginalization, barriers to services, *etc*.) [7,8,9,10]. These conditions often lead to premarital or extramarital sex occurring frequently in this population [10,11,12]. Therefore, rural migrants might be a population at high risk of HIV infection [13,14,15], but there is a lack of international studies assessing this subject.

To control the rate of HIV/AIDS infection in the general population, effective behavioral intervention programs are provided to migrants [16]. Among these, community-based models are widely used [17,18,19], and their efficacy has been confirmed in preliminary investigations in China [20,21,22]. Community-based models are flexible and do not cost much, making them particularly useful for rural migrants who work long shifts for low wages. However, the coordination of the Community Health Agency (CHA) provision of HIV/AIDS prevention has not yet been established in northwestern China. Currently, HIV/AIDS prevention efforts are carried out by limited number of people from the Center of Disease Control (CDC), which conducts short-term, small-scale and superficial activities [23]. In addition, the seasonal migration of rural migrants plays a significant role in the impact of HIV/AIDS prevention. It is necessary to construct a management network of HIV/AIDS prevention for the migrants, which unfortunately is still missing. However, a family planning network for migrants has already been established, which includes HIV/AIDS prevention efforts [15].

The objective of the present study was to measure the efficacy of a HIV/AIDS prevention and control program for rural migrants in the Shaanxi Province. Participants were divided into an experimental (EG) and a control group (CG). We analyzed their HIV/AIDS-related knowledge, attitude and behavior (KAB) before, during and after the intervention at both rural and urban sites. We hypothesized that our coordinated intervention at their home village and place of work greatly improve participants’ HIV/AIDS-related KAB. Results of the present study might also be applicable to migrant populations elsewhere in the world.

## 2. Methods

### 2.1. Study Design

**Figure 1:**
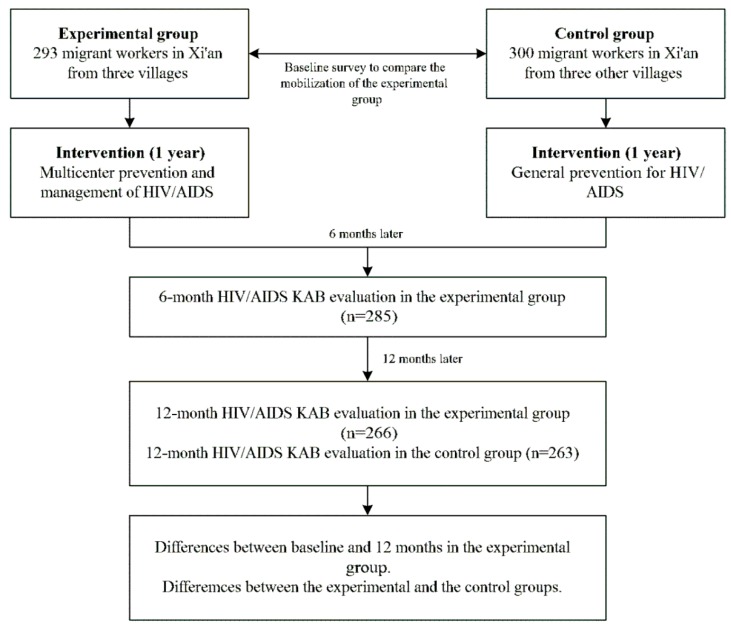
Participants’ flowchart.

The design of our intervention is shown in Figure 1. A quasi-experimental study was conducted between March 2009 and April 2010. In the study, six villages were selected, based on the pre-study assessment of KAB on HIV/AIDS. A year-long multicenter prevention intervention was conducted at Xi’an and the home villages of those participants assigned to the EG. The CG received no intervention, other that the general intervention provided in the general population in China. The effects were evaluated after 6 and 12 months of intervention. The KAB scores of the EG and CG were assessed and compared (Figure 1).

### 2.2. Study Site

We chose one of the poorest counties in China: Lantian County in Shaanxi Province [24]. Most of the young adults from the Lantian County work in Xi’an, the capital city of the Shaanxi Province [21]. They regularly return home for long holidays or for planting and harvesting seasons [21].

### 2.3. Participants

Inclusion criteria were: (1) 18–49 years old; (2) household registration in rural Lantian; (3) working in a fixed location in downtown Xi’an for at least three months; (4) normal development and language; and (5) willing to participate in this study. 

Rural migrants with similar geographical or blood relationships tend to work together. Therefore, 293 migrants in three villages of Lantian County (Mandao, Mengyan and Guizhang) who work in five fixed communities in Xi’an were assigned to the experimental group (EG). The remaining 300 migrants, from other villages of similar background (Songjiamiao, Songjia and Qingyangzhuang, all from Lantian County), were assigned to the control group (CG). The protocol was reviewed and approved by the Human Research Ethics Committee of the Xi’an Jiaotong University College of Medicine, and all participants provided written informed consent.

### 2.4. Interventions

The education was tailored to each individual on the basis of the assessment of one’s knowledge about HIV and the capacity to accept the knowledge, and the professional intervention was implemented by the entire intervention group for each individual. The intervention was carried out at least once a month to ensure that the knowledge and methods were taught step by step. Each intervention was based on the examination of the contents of the previous intervention. The content was not repeated if it was mastered, and misunderstood content was corrected; meanwhile, new content was taught. 

The intervention team included the CDC staff, family planning workers, community health workers and assistants, all with their specifics tasks. The CDC staff was mainly there to train and guide the other members of the intervention team, to provide HIV/AIDS-related knowledge and skills, and to perform HIV testing. In China, family planning workers are in charge of the birth system; their tasks were to perform physical examinations and to assist the community health workers in carrying out HIV/AIDS-related knowledge publicity, and in teaching condom use methods. Community health workers were the implementers of the HIV/AIDS-related knowledge education, and performed counseling psychology, basic physical examinations, education on sexual partner’s communication, and condom use teaching. The other team members were assistants, and were in charge of following up migration population, evaluating intervention’s effect, and assisting the community health workers to carry out health education. 

#### 2.4.1. Framework of Intervention

**Figure 2:**
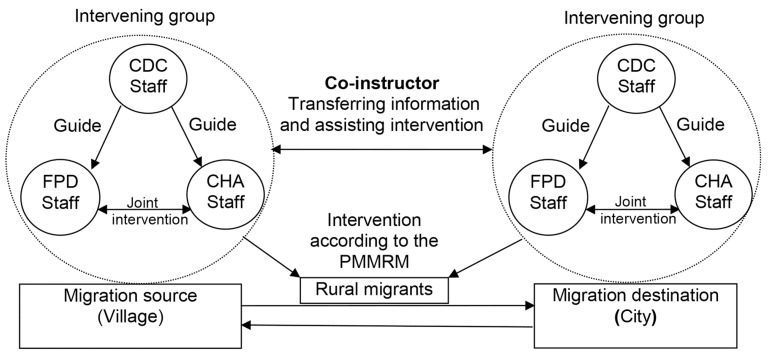
Framework of intervention.

The 12-month intervention program includes four aspects (Figure 2). (1) The family planning network of rural migrants provides an information-exchange platform. Prevention education is conducted by coordinating services and education between the city (migration destination) and the rural area (migration source); (2) A collaborative intervention group was established between the cities and villages, including staff from the CDC, FPD and CHA. Community-based intervention was also implemented; (3) In order to ensure smooth communication and effective implementation, five professionals (co-instructors) were sent to the five communities in Xi’an. These co-instructors were responsible for objective tracking, health education and intervention evaluation. In addition, when the migrants returned home during the planting and harvesting seasons, three of the five professionals went to three villages to help with the work;(4) To avoid duplication, the “HIV/AIDS Prevention Management Manual for Rural Migrants” (PMMRM) was drafted and distributed to each migrant to maintain a standardized and systematic intervention [21]. Members of the intervention team conducted a dynamic and systematic management in accordance with the PMMRM.

#### 2.4.2. HIV/AIDS Prevention Management Manual for Rural Migrants

The PMMRM is divided into three parts. (1) Basic information, including the name, age, home address, telephone numbers. The contact information for village health clinics and urban community health centers are also included; (2) The intervention procedure, divided into assessment, survey, implementation, and evaluation. The assessment framework is guided by the KAB theory, including the KAB and its influencing factors, physical assessment (focusing on the reproductive system) and laboratory examination results. The questions based on physical assessment also include the knowledge, attitude, behavior and physical characteristics. The signatures from investigators and surveyed subjects were required to confirm the time, location, content, methods, and names of investigators and migrants in order to supervise the intervention group; (3) The appendix, which contains the basic skills for preventing HIV/AIDS, using texts and pictures. 

#### 2.4.3. Intervention Methods

The project meeting was held in the village during the Spring Festival 2009, when migrants returned home. The participants included: co-instructors, rural migrants and their family members. During the meeting, the research purpose, significance and methods were introduced. The participants provided written informed consent, and the PMMRM was distributed. Intervening staff recorded the date at which migrants left for Xi’an, their employer’s address, and their contact information. Before the migrants left, the staff of the migration source area reminded them to take the PMMRM, and informed the intervening staff of the migration destination area. Within 1 week of the migrants arriving in Xi’an, the city’s intervening staff contacted them. Subjects in the EG groups met the intervening staff once a month.

Participants in the CG received no specific HIV/AIDS education, except what they may have learned from general education provided to the general population on billboards, pamphlets or other promotional material.

### 2.5. Measures

The effects of the intervention were measured by the questionnaire of KAB on HIV/AIDS for migrants [25,26,27]. Based on the “HIV/AIDS knowledge, attitude scale” of the World health Organization (WHO) and an extensive literature review, the questionnaire was revised in order to consider the migrants’ low education level and the AIDS-related behavioral characteristic sheet. Participants completed all questionnaires by themselves; if they were unable to do so, an investigator explained all questions and filled the questionnaires based on the participant’s responses.

#### 2.5.1. Questionnaires

The questionnaires included demographic data (gender, age, education, and marital status), and four statements about HIV/AIDS (25 items: 2 about basic knowledge, 8 about transmission routes, 9 about transmission misconceptions, and 6 about prevention and treatments). All items had three possible responses: “True, Don’t know and False”. Positive statements were scored as “True = 2, Don’t know = 1 and False = 0”, and negative statements are scored as “True = 0, Don’t know = 1 and False = 2”. The total score ranges from 0 to 50. The higher the score, the better the knowledge level of HIV/AIDS is. Attitude: The HIV/AIDS attitude questionnaire consists of 3 dimensions (attitude to AIDS: 7 items; attitude to the related behavior: 6 items; attitude to the infected patient: 13 items) and 26 questions with a 4-point scale ranging from “agree” to “disagree”. There are 11 positive items scored as “agree = 3, undecided = 2, indifferent = 1, disagree = 0” and 15 negative items reverse-scored. The total possible score ranges from 0 to 78. Higher score shows more positive attitude. Sexual behavior: the behavior questionnaire consists of 16 items, in which 8 are related to sexual behavior, 4 to selling blood, and 4 to drug abuse.

#### 2.5.2. Reliability and Validity of the Questionnaire

The English version of the questionnaire was back-translated and discussed repeatedly by linguists. Before use, the questionnaire was assessed thrice by three experts, who majored in epidemiology, communicable diseases and nursing, respectively, to ensure the format and content validity of the questionnaire. Furthermore, 50 subjects were pre-surveyed. The Cronbach’s alpha coefficient of the knowledge questionnaire was 0.79, 0.78 for the attitude questionnaire, and 0.81 for the whole questionnaire.

### 2.6. Power and Statistical Analysis

In the present study, sample size determination was based on the rate of change in high-risk sexual behaviors after intervention. It was reported that the high-risk sexual behavior of rural migrants changes from 0.3% to 18.2% after comprehensive intervention [1,28]. Thus, based on the initial results of our survey, the condom use rate in each high-risk sexual behavior of migrant workers was expected to increase from 4.5% to 14.5%. Therefore, using a 90% confidence and an α of 0.05, the sample size required for each group of this study was 225 migrants (total of 450). Taking into account a 30% drop-out rate, the required sample size was 586 migrants (293 migrants/group).

Statistical analysis was performed using SPSS 16.0 for windows (SPSS Inc., Chicago, IL, USA). All variables were initially analyzed descriptively, including means and standard deviation for the quantitative data, and frequencies and percentages for the categorical variables. The difference in knowledge or attitude between the EG and CG was assessed by independent sample t-test. The chi-square test was used to detect the difference of behavior on HIV/AIDS. *P*-values < 0.05 were considered statistically significant.

## 3. Results

Figure 1 shows the participants’ flowchart: 293 EG migrants and 300 CG migrants completed the baseline survey. After 6 months intervention, 8 migrants from the EG were lost to follow-up due to change of employer. In the following 6-months an additional 19 migrants were lost to follow-up. After 12 months, the EG consisted of 266 participants and the CG of 263 participants.

There was no significant difference in age, gender, marital status, and education between the EG and CG (*p* > 0.05). Most rural migrants were males (53.2% and 56.0% in EG and CG), married (91.8% and 92.3% in EG and CG), and had completed junior middle education level (68.3% and 66.3% in EG and CG) (Table 1).

**Table 1:** Baseline characteristics of participating rural migrants.

Demographic information	EG	CG	*x*^2^/*t*	*P*
	(*N* = 293)	(*N* = 300)		
**Age (mean ± SD)**	36.56 ± 9.01	36.67 ± 8.96	−0.145	0.885
**Gender (*%*)**				
Male	53.2	56.0	0.500	0.510
Female	46.8	44.0		
**Marriage status (*%*)**				
Married	91.8	92.3	2.829	0.243
Non-married	7.5	7.7		
Divorced/windowed	0.7	0.0		
**Education (*%*)**				
Primary or below	8.5	5.7	3.140	0.208
Junior middle school	68.3	66.3		
Senior middle school/occupation school	23.2	28.0		

Abbreviations: EG = experimental group. CG = control group.

### 3.1. HIV/AIDS Knowledge

**Figure 3:**
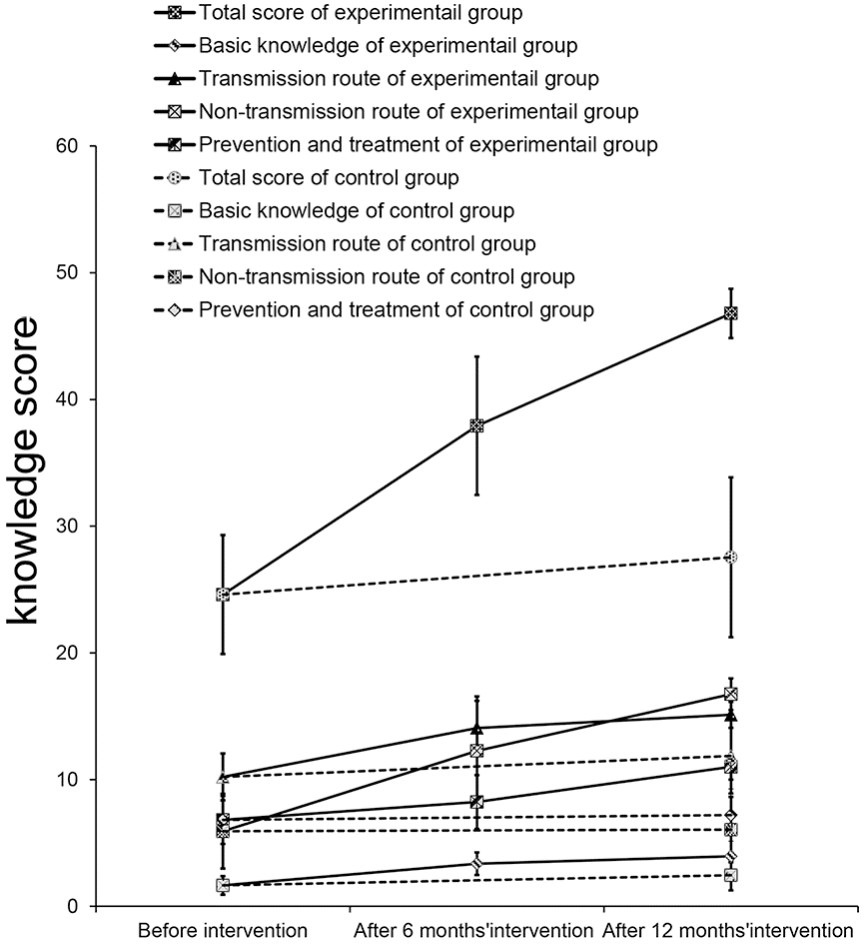
Changes in knowledge scores.

Table 2 shows the HIV/AIDS knowledge scores of participants. Before the intervention, there was no significant difference in the total score and each dimension score between the EG and CG (*p* > 0.05). Aside from the relatively high score on transmission route knowledge, the total score and the other dimension scores were low. The score for misconceptions about transmission route knowledge was especially low. There were significant differences between the EG average knowledge score before and after 6 or 12 months of intervention (*p* < 0.05). During the intervention, the total score and each dimension score gradually increased, and the scores after 12 months were close to the maximum value (Figure 3). The total score and dimension scores of the EG were significantly higher than those of the CG after 12 months of intervention (*p* < 0.05). In the CG, total score, basic knowledge score and transmission route score after 12 months were significantly higher than those before the intervention (*p* < 0.05), but there was no significant increase in the scores for the categories “misconceptions about transmission route” and “prevention-treatment” after the intervention period (*p* > 0.05).

### 3.2. HIV/AIDS Attitude

**Figure 4:**
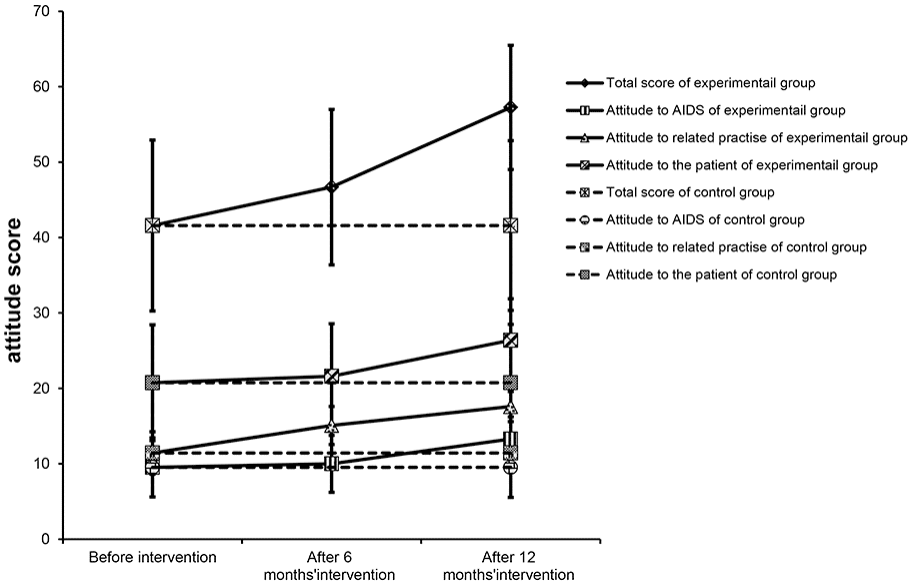
Changes in attitude scores.

Table 3 shows the scores about attitude on HIV/AIDS. Before the intervention, there were no significant differences in the total attitude score and each dimension score between the EG and CG (*p* > 0.05). After 6 months, the EG total score and sub-attitude scores relating to behavior changes were significantly increased (*p* < 0.05). However, the scores of attitude to AIDS and infected patients did not change (*p* > 0.05). After 12 months, the total score and each sub-score of the EG were significantly higher than those before intervention or those of the CG (*p* < 0.05); the scores also gradually increased with prolonged intervention time (Figure 4). In the CG, the total score and each sub-score after 12 months of intervention showed no significant difference compared with those before intervention (*p* > 0.05).

### 3.3. HIV/AIDS Behavior

Before the intervention, only one participant sold blood or accepted voluntary HIV counseling and testing (VCT). Therefore, the comparison of HIV/AIDS behavior focused on sex-related behaviors. Excluding the subjects who had no sexual activity during the last 3 months, there were 274 (pre-intervention), 280 (post-6 months intervention), and 266 (post-12 months intervention) subjects in the EG, and 286 (pre-test) and 260 (post-test) in the CG in whom sex-related behaviors were compared.

Table 4 shows the comparison of HIV/AIDS-related sexual behaviors. Before the intervention, there were no significant differences between the EG and CG (*p* > 0.05). After 6 months, there were significant changes in only 2 items in the EG (“communicate with sexual partner” and “test rate of STD”) (*p* < 0.05). After 12 months, we found that most behaviors (commercial sex, use of condom, reason for using condom, *etc.*) were significantly improved (*p* < 0.05), and that only one item (“number of sexual partners”) did not improve significantly (*p* > 0.05). For each item within the CG, there was no significant difference between pre- and post- intervention (*p* > 0.05).

## 4. Discussion

We explored the efficacy of a HIV/AIDS prevention and control program for rural migrants in the Shaanxi Province by analyzing the HIV/AIDS-related KAB variations resulting from interventions at both rural and urban sites. Comparison between the EG and CG highlighted that the intervention achieved a significant improvement of participants’ HIV/AIDS-related KAB.

There are many studies on HIV/AIDS prevention in rural migrants, but most interventions are cross-sectional and one-sided [29,30]. In the present study, intervention evaluation was carried out at different times at both the migration source and destination, in order to ensure the continuity of the education.

**Table 2:** Differences in HIV/AIDS knowledge scores.

HIV-Related Knowledge	EG	EG	EG	CG	CG	t**				
	Before Intervention	After 6 months’ Intervention	After 12 months’ Intervention	Before Intervention	After 12 months’ Intervention					
	1	2	3	4	5	1–4	1–2	1–3	3–5	4–5
Total score for knowledge	24.60 ± 4.69	37.92 ± 5.46	46.79 ± 1.94	24.96 ± 4.46	27.55 ± 6.31	−0.946	−31.407 *	−74.189 *	47.290 *	−5.553 *
1-Basic knowledge	1.65 ± 0.72	3.36 ± 0.89	3.95 ± 0.28	1.69 ± 0.68	2.45 ± 1.19	−0.666	−25.375 *	−50.568 *	19.872 *	−9.195 *
2-Transmission route	10.21 ± 1.85	14.07 ± 2.14	15.11 ± 1.03	10.31 ± 1.65	11.86 ± 2.96	−0.686	−23.182 *	−39.233 *	16.823 *	−7.523 *
3-Misconceptions about transmission route	5.92 ± 2.95	12.27 ± 4.29	16.74 ± 1.24	5.92 ± 2.92	6.05 ± 2.58	0.034	−20.646 *	−57.411 *	60.753 *	−0.557
4-Prevention and treatment	6.82 ± 1.90	8.23 ± 2.13	11.00 ± 1.00	7.05 ± 1.85	7.20 ± 2.07	−1.475	−8.372 *	−32.893 *	26.870 *	−0.914

Abbreviations: EG = experimental group. CG = control group. Data are presented as (mean ± SD).*p < 0.05; ** Comparisons between different pairs of measurements.

**Table 3:** Differences in HIV/AIDS attitude scores.

HIV-Related Attitude	EG	EG	EG	CG	CG	*t* **				
	Before Intervention	After 6 months’ Intervention	After 12 months’ Intervention	Before Intervention	After 12 months’ Intervention					
	1	2	3	4	5	1–4	1–2	1–3	3–5	4–5
Total score of attitude	41.59 ± 11.33	46.69 ± 10.31	57.26 ± 8.23	43.07 ± 10.35	41.47 ± 11.25	−1.653	−5.659 *	−18.818 *	18.408 *	1.756
1-Attitude to AIDS	9.52 ± 3.92	10.00 ± 3.78	13.28 ± 2.94	10.11 ± 3.37	9.83 ± 3.97	−1.889	−1.512	−12.903 *	11.354 *	0.922
2-Attitude to related behaviors	11.43 ± 2.81	15.08 ± 2.52	17.59 ± 2.00	11.76 ± 2.45	11.38 ± 2.55	−1.881	−16.894 *	−30.537 *	31.131 *	1.802
3-Attitude to the patient	20.75 ± 7.67	21.61 ± 6.95	26.39 ± 5.48	21.19 ± 7.24	20.26 ± 7.74	−0.728	−1.411	−10.074 *	10.495 *	1.474

Abbreviations: EG = experimental group. CG = control group. Data are presented as (mean ± SD). * *p* < 0.05; ** Comparisons between different pairs of measurements.

**Table 4:** Differences in HIV/AIDS-related behavior.

HIV-Related Behavior	EG	EG	EG	CG	CG	*χ*^2^ **				
	Before Intervention	After 6 months Intervention	After 12 months Intervention	Before Intervention	After 12 months Intervention					
	1	2	3	4	5	1–4	1–2	1–3	3–5	4–5
**Number of sexual partners**										
1	251 (91.6)	264 (94.3)	249 (93.6)	259 (90.6)	245 (94.2)	0.712	4.521	5.546	2.498	3.421
≥2	19 (6.9)	16 (5.7)	17 (6.4)	20 (7.0)	13 (5.0)					
0	4 (1.5)	0 (0.0)	0 (0.0)	7 (2.4)	2 (0.8)					
**Participate in commercial sex**										
Yes	20 (7.3)	25 (8.9)	7 (2.6)	14 (4.9)	9 (3.5)	2.058	0.896	12.113 *	14.071 *	3.505
No	250 (91.2)	249 (88.9)	259 (97.4)	265 (92.7)	238 (91.5)					
Not clear	4 (1.5)	6 (2.1)	0 (0.0)	7 (2.4)	13 (5.0)					
**Condom Use**										
Never	169 (61.7)	163 (59.5)	132 (57.1)	167 (58.4)	147 (56.5)	2.448	0.976	9.477 *	10.778 *	0.396
Sometimes	93 (33.9)	94 (34.3)	111 (36.8)	111 (38.8)	107 (41.2)					
Always	12 ( 4.4)	17 ( 6.2)	23 ( 6.0)	8 ( 2.8)	6 (2.3)					
**Reason for condom use**										
Contraception	91 (85.0)	91 (82.0)	74 (64.9)	100 (84.0)	98 (86.7)	0.144	0.371	11.827 *	14.708 *	0.336
Prevention of diseases	16 (15.0)	20 (18.0)	40 (35.1)	19 (16.0)	15 (13.3)					
**Communication with sexual partners to encourage condom use**										
Never	43 (89.6)	33 (62.3)	16 (34.8)	53 (81.5)	48 (80.8)	1.440	10.709 *	30.544 *	22.262 *	0.799
Sometimes	4 (8.3)	16 (30.2)	18 (39.1)	9 (13.8)	7 (11.7)					
Always	1 (2.1)	4 (7.5)	12 (26.1)	3 (4.7)	5 (8.3)					
**STD infection **										
Yes	2 (0.7)	8 (2.9)	8 (3.0)	4 (1.4)	6 (2.3)	2.977	10.082 *	14.896 *	8.734 *	0.650
No	264 (96.4)	270 (96.4)	258 (97.0)	275 (96.2)	248 (95.4)					
Not clear	4 (1.5)	0 (0.0)	0 (0.0)	1 (0.3)	1 (0.4)					
Missing value	4 (1.5)	2 (0.7)	0 (0.0)	6 (2.1)	5 (1.9)					
**VCT conducted**										
Yes	0 (0.0)	4 (1.4)	17 (6.4)	0 (0)	1 (0.4)		2.202	18.080 *	14.352 *	1.486
No	274 (100.0)	276 (98.6)	249 (93.6)	286 (100.0)	259 (99.6)					

Abbreviations: EG = experimental group. CG = control group. STD = sexually transmitted disease. VCT = voluntary HIV counseling and testing. Data are presented as *n* (%). * *p* < 0.05; ** Comparisons between different pairs of measurements.

Secondly, the independent AIDS-PMMRM was provided for each migrant. Based on systematic assessment, each intervention was implemented in accordance with migrant’s physical and psychological characteristics and lifestyle, to ensure that each participant receives the appropriate educational content. This was done by tailoring the intervention to the participants according to his sexual behaviors (low- *vs.* high-risk), to his literacy, to his technology accessibility (compact disc *vs.* brochure) and to his availability. In addition, studies showed that HIV/AIDS prevention knowledge from different sources will produce different results, and that people are most willing to accept the prevention knowledge from medical professionals [31,32]. In this study, a professional team administered the intervention. The staff from the CDC was mainly responsible for instructing interventions with professional knowledge, and the staff of the CHA and family planning services conducted direct interventions due to their strong social abilities. The role of community organizations in HIV/AIDS prevention has been recognized [33]. With repeated intervention, the knowledge of the participants increases. Meanwhile, the capability of the medical staff also increases. 

Compared with the EG, migrants in the CG had improved HIV/AIDS “basic knowledge” and “transmission routes” after 12 months, while the other aspects (“misconceptions about transmission routes” and “prevention and treatment”) were not improved. These results indicate that the HIV/AIDS propaganda administered in urban and rural areas have a certain impact, but the breadth and depth of the effects are insufficient as a consequence of complex factors, such as time, place, method, educators, *etc.* It also suggests that exploring a scientific, systematic and feasible intervention program is necessary.

The level of HIV/AIDS knowledge directly affects the attitude to AIDS, and participants with a lower cognitive level have more serious discriminatory attitudes towards those suffering from HIV/AIDS [12,34]. Moreover, attitude is influenced by personal experience, values, and emotions, and changing these attitudes takes a long time. In the present study, the influencing factors on psychology and society are accounted for during the circular assessment and intervention. For instance, in the assessment of the migrants at the migration source, we observed that “subjunctive community” (composed by migrants’ social network) [35] and the “key person” play a very important role in the ideas of the group.The researchers purposely arranged for the “key person” to communicate with the HIV-positive migrant, and encouraged them to exploit the “subjunctive community” in peer education. Simultaneously, in rural migration destinations, education is promoted by social and family support.The information exchange and emotional communication among family members has an important effect on individual perceptions, beliefs, and behaviors [36].Intervention of family members can not only provide migrants with a more powerful support system, but also acts to reduce the possibility of HIV transmission from migrants to their family members.

After 6 months of intervention, the participants recognized that condoms are a simple and efficient mean to prevent HIV/AIDS, and they learned some skills for communicating with their sexual partners. The reason why the detection of STDs increases is that more migrants are tested. After 12 months, the rates of condom use within the EG were significantly increased. Also, more migrants used condoms for the purpose of disease protection. The incidence of participation in commercial sex also decreased, but the number of sexual partners did not change. We concluded that participants’ understanding of sexual health and the extent to which they trust their sexual partners influenced their sexual behavior. This conclusion is in accord with previously published research [37]. Commercial sexual partners have been recognized as the most dangerous population in the spread of HIV/AIDS, while stable partners are safer as a consequence of comprehensive understanding and trust [15,38,39]. The results achieved may also depend upon intervener’s skills. A previous study showed that an 18 months practice was needed to improve health prevention staff’s skills [19]. In our study, the conductors implemented the HIV/AIDS prevention mode for only 1 year. Their intervention skills would therefore need to be improved, mainly because sexual behavior is a complex behavior influenced by social, emotional and situational factors.

After 12 months, the frequency of VCT in the EG were significantly increased. Regular tracking and information collection showed that those who participated in VCT were mainly those who have sold blood or transfused blood, while drug users and people with dangerous sexual behavior still rarely participated in VCT. This means that participation in VCT may be related to the routes of infection. If the route of infection involves what is traditionally considered to be "immoral behavior", infected individuals are less willing to participate in VCT, fearing discrimination and apathy.

In the present study, we selected rural migrants aged 18–49 because: (1) migrants aged 18–49 represent 69.9% of all migrants in China [6]; and (2) a report on the 2010 situation of the AIDS epidemic in the Shaanxi province showed that more than 80% of AIDS-infected people and patients are 18–49 years old [4]. Finally, a previous survey showed that in migrants aged 18–49, 62.3% had a high-risk sexual behavior. However, more studies are needed to assess the complete population of migrants in China and in other countries.

The present study suffers from some limitations. First, the home villages of the participants were relatively close to their working place. Considering that most people in the Lantian County worked in Xi’an city and also in order to facilitate the survey, the Lantian County and Xi’an city were selected as survey locations in this study. Xi’an is the capital city of Shaanxi Province while Lantian County is in the territory of Shaanxi Province. Previous studies have demonstrated that rural migrants working in places farther from home have the higher incidence of high-risk behavior compared with those working closer to home [13,14,15]. Therefore, in the present study, the incidence of high-risk behavior in the migrants was clearly lower than that reported in migrants working in Shanghai, Beijing, or Guangzhou. Second, the dropout rate of participants was high. Since the network platform about the information on migrants is not fully developed, five co-instructors were sent to track the migrants. However, during the year of intervention, about 10% dropout still occurred in the EG and CG. Hence, an appropriate migrants’ management network has to be established before the program for HIV/AIDS prevention for the migrants may be implemented to the whole population. Finally, the long-term effects of the intervention need further evaluation. After one year of intervention, the attitudes and behaviors of migrants toward AIDS were improved. However, whether these knowledge, attitudes and behavior could be retained or improved after one year needs to be verified in a follow-up study.

## 5. Conclusions

We observed that the cross-center HIV/AIDS prevention and management model could improve rural migrants’ HIV/AIDS-related knowledge, attitude and behavior . Further studies would further illuminate the extent of this improvement, examine how it may be maintained, and study the impact of this intervention on the HIV acquisition rate of participants. In addition, this model could be used elsewhere in the world.
